# Sclerotherapy for the recurrent granulomatous epulis with pingyangmycin

**DOI:** 10.4317/medoral.21422

**Published:** 2017-02-04

**Authors:** Yu Cai, Rui Sun, Ke-Fei He, Yi-Fang Zhao, Ji-Hong Zhao

**Affiliations:** 1 The State Key Laboratory Breeding Base of Basic Science of Stomatology (Hubei-MOST) & Key Laboratory of Oral Biomedical Engineering of Ministry of Education, School & Hospital of Stomatology, Wuhan University, Wuhan, 430079, P. R. China; 2 Department of Oral and Maxillofacial Surgery, School and Hospital of Stomatology, Wuhan University, Wuhan, 430079, P. R. China

## Abstract

**Background:**

Relapse of granulomatous epulis is common after surgery because of local irritations, hormonal level *in vivo*, or incomplete resection. Currently, if recurrence occurs, then extraction of the teeth adjacent to the lesion is commonly performed, which may influence the aesthetics or masticatory function. Thus, a more effective and less aggressive treatment method is urgently demanded, particularly for the recurring lesion. This study investigated the effects of the intralesional pingyangmycin (PYM) injections for the recurrent granulomatous epulis and assessed the complications.

**Material and Methods:**

A total of 16 patients with recurrent granulomatous epulis underwent intralesional PYM injections, between July 2010 and June 2014. The effects and complications of the treatment were retrospectively reviewed.

**Results:**

The total number of injections performed was 48 (for all patients). The median number of injections per patient was three (range, two to four). All cases completely recovered with no recurrence and resorption of the alveolar bone after a follow-up of more than 12 months. The complications included slight bleeding, local swelling and pain following injection. All these symptoms resolved 7 to 10 days after the injection.

**Conclusions:**

In summary, intralesional PYM injections may be a preferred option for recurring granulomatous epulis.

**Key words:**Granulomatous epulis, recurrence, pingyangmycin, sclerotherapy.

## Introduction

Epulis is a clinically diagnostic term referring to a reactive focal connective tissue proliferation in the gingiva, and its exact histological nature is unknown. Epulis occurs at any gender and age, but is more common in female and young people (1). Although epulis has different classifications in the literature, the most widely accepted classification in China divides epulis into three main types by their tissue origin, namely, granulomatous epulis (epulis haemangiomatosa), fibrous (fibroid) epulis and giant cell (myeloid) epulis (2).

Granulomatous epulis, a smooth or lobulated exophytic lesion with a deep red or purplish colour (3), is also referred to as gingival pyogenic granuloma(4,5), lobular capillary haemangioma of the gingiva (3), and epulis granulomatosa (6). Local irritants, such as calculus, hormonal factors, certain drugs, and poor oral hygiene, may contribute to the development of granulomatous epulis (5). The management of granulomatous epulis depends on the clinical manifestations. Removal of the causative irritants, clinical observation and follow-up may be suggestive when the lesion is small, painless and free of bleeding. Although conservative excision, which extends down to the periosteum and reserves the teeth, was the usual treatment, invasive resection, which includes removing the adjacent teeth, should be performed to treat the extensive lesion with serious loose tooth or the recurrent lesion (3).

Therefore, more effective and less aggressive treatment methods should be discovered for the recurrent granulomatous epulis. In this study, the intralesional pingyangmycin (bleomycin [BLM] A5 hydrochloride, PYM) injection was used to treat the 16 patients with recurrent granulomatous epulis. All cases completely recovered without recurrence, which indicated that PYM sclerotherapy may be an additional treatment method for the recurrent granulomatous epulis.

## Material and Methods

Between July 2010 and June 2014, 16 patients with recurrent granulomatous epulis were prospectively enrolled for the intralesional PYM solution injection treatment at the Department of Oral and Maxillofacial Surgery, Hospital of Stomatology, Wuhan University (see  Table 1 for the clinical data). This series comprised 0 males and 16 females, whose ages ranged from 15 years to 38 years, with a median age of 26.5 years. Furthermore, all of the patients had normal menstrual cycles, had a medical history that revealed no presumable cause, and had no history of drinking alcohol or smoking. Informed consent was obtained from all instances. The study was approved by the review board of the Ethics Committee of the Hospital of Stomatology, Wuhan University.

**Table 1:**
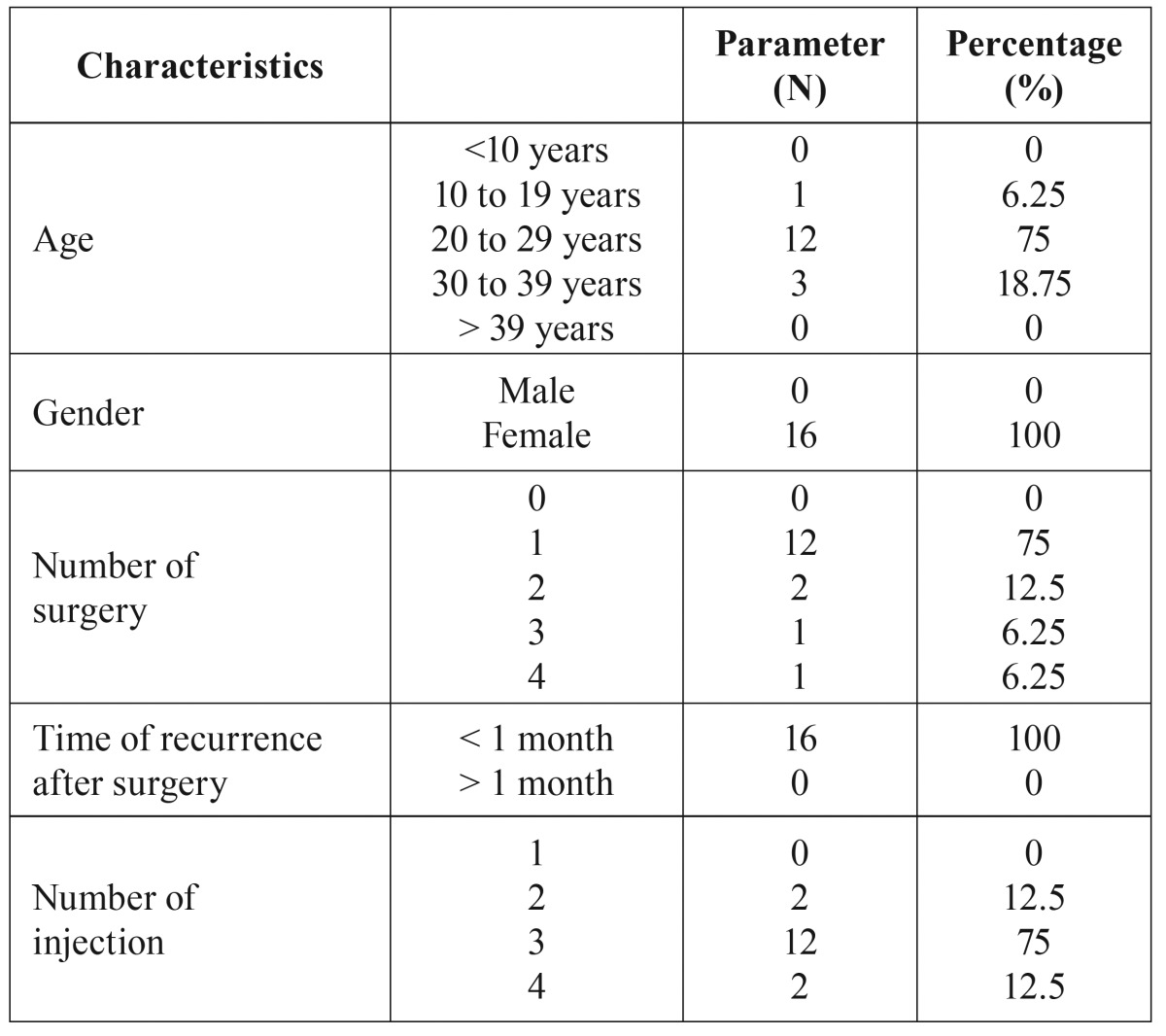
Clinical data of patients with the recurrent granulomatous epulis.

**Figure 1:**
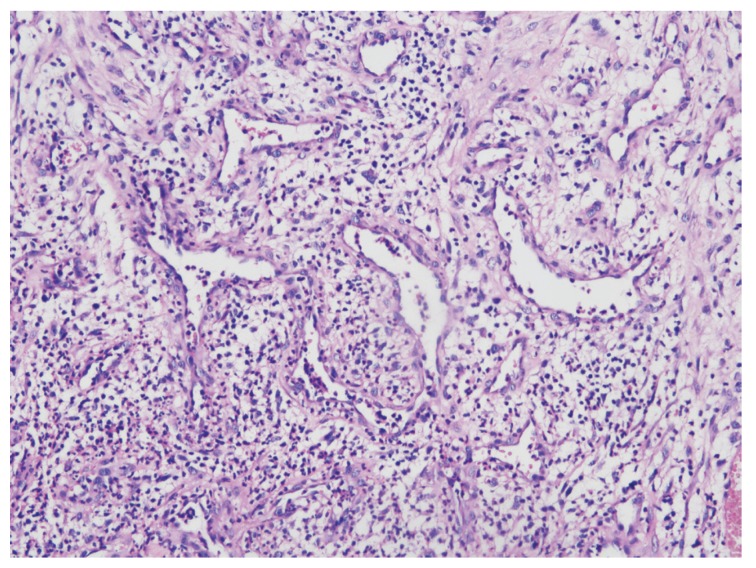
The microscopic examination (40×) of the specimens shows the chronic inflammatory granulation tissue with increased vascularity.

* Inclusion criteria

1. The clinical diagnosis of all cases was epulis when the patients first visited the department.

2. All these cases first were admitted to the department. Extensive resection, including removal of the periodontium, periosteum, some alveolar bone, and irritants around the lesion, was performed. Then, adjacent gingival flap was used to close the wound, regardless of whether it occurred for the first time or recurred after surgery in other hospitals. In addition, the involved teeth were removed if the tooth mobility index was greater than 2 during surgery;

3. The histopathologic examination of the all specimens after surgery confirmed the diagnosis of granulomatous epulis (Fig. 1).

* Exclusion criteria

1. Patients with long-term pharmacotherapy (phenytoin, cyclosporine, or nifedipine).

2. Pregnant patients.

3. Lesions that were definitely caused by foreign material.

* Approach and technique

After recurrence, all patients were recommended to receive intralesional PYM injection. All patients with follow-up of more than 12 months after the final treatment were included in this study.

PYM has been described previously (7). After the regional anaesthesia, PYM solution (concentration, 2 mg/mL) was injected into the pedicel until the lesions slightly bled. A single dose of 3 mg per session was not exceeded. After injection, the patients were carefully observed for resolution. The injections were repeated seven days later if necessary.

## Results

**Figure 2:**
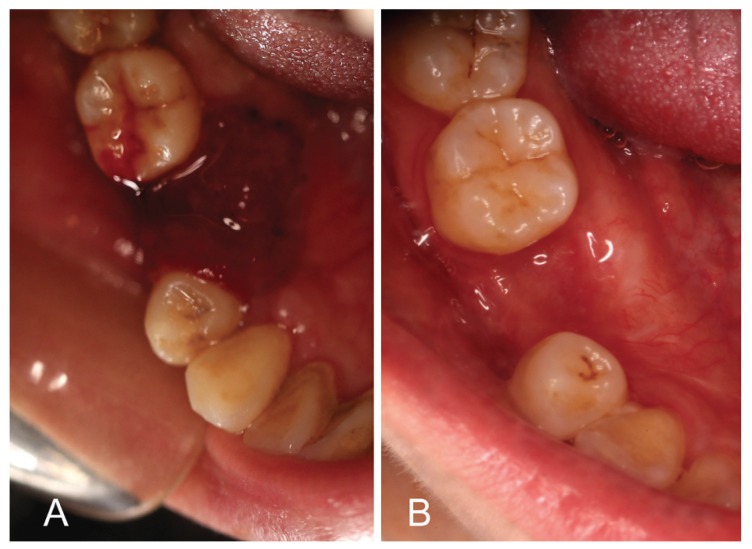
A) A bright red, easily haemorrhage and well-defined tumor at the gingiva of the right lower second premolar was relapsed after surgery. (B) The lesion was disappeared after four times intralesional injection of PYM.

Among the 16 cases, 12 cases experienced one surgery, 4 cases underwent multiple surgeries, and 7 cases had tooth extraction. All of the lesions relapsed no more than one month after the surgery. The total number of injections performed was 48. The median number of injections per patient was three (range, two to four). The median duration of follow-up was 18 months (range, 12 months to 24 months). Overall, in this series, all of the patients completely recovered with no recurrence (Fig. 2). No difference was evident in the response rate to PYM injection between patients who had undergone previous tooth extraction and those who had not.

**Figure 3:**
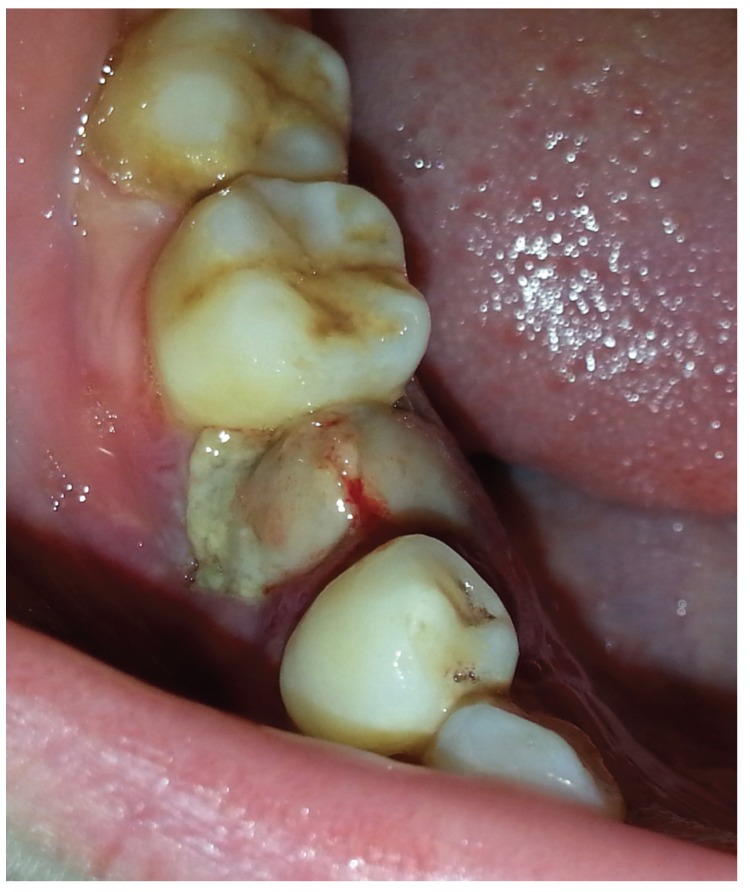
The ulceration was found on the surface of the lesion about three days after injection.

Small haemorrhage occurred on the surface of the lesion after injection, which stopped bleeding for no more than 1 h. Slight swelling and pain occurred around the lesion on the second day following sclerosant injection;these symptoms resolved in 7 to 10 days. Approximately three days after injection, ulceration was observed on the surface of all of the lesions (Fig. 3), which vanished along with disappearance of the entire lesion. No other complications were associated with the injection.

## Discussion

It’s widely accepted that epulis is not the true neoplasm but the hyperplastic lesion, which is the reason why epulis is not referred by the World Health Organization Classification of Tumours. Therefore, the classification and terminology of epulis are conflicting in the literature. In 2004, Sapp divided epulis into four main kinds, namely, peripheral fibroma, peripheral ossifying fibroma, pyogenic granuloma (including pregnancy tumour) and peripheral giant cell granuloma, based on the histological feature. However, other literature classified epulis into acanthomatous epulis, giant-cell epulis, ossifying epulis and periodontal fibromatous epulis. In 2008, the Dental Dictionary categorised epulis into congenital of newborn, epulis fissuratum, epulis-giant cell, and epulis granulomatosa. Furthermore, some categorizsation was also based on the age group. These different classifications result in the confusing diagnoses and treatments.

Therefore, Chinese histopathologists and doctors (1) summarizsed the various classifications of epulis in the literature and renamed the terms as follows: fibrous (fibroid) epulis, granulomatous epulis (epulis haemangiomatosa), and giant cell (myeloid) epulis (2), which is a straightforward nomenclature to manifest the histological type and pathogenic site. This classification was most widely accepted and was referred in the textbook of Oral and Maxillofacial Surgery in China.

Granulomatous epulis, which is also referred to as gingival pyogenic granuloma, is the most common disease among oral pyogenic granuloma (3). Although the factors that influence the development, growth rate and tendency of granulomatous epulis recurrence are still unknown, local irritations (e.g. dental plaque, calculus, foreign material within the gingival crevice or injury), pharmacotherapy (phenytoin, cyclosporine or nifedipine) and hormonal status (estrogen or pregnancy) should be considered when making the treatment plan (2,3).

With regard to the treatment, a comprehensive treatment is the strategy to cure granulomatous epulis, generally including aetiological treatment (removal of stimulus) and surgical procedures (excision and biopsy of the lesion). However, the argument about treatment focuses on removing the teeth involved by the lesion, some clinicians considering the high rate of recurrence suggest that removing the lesion and the involved teeth together (1), others recommend to reserve teeth in consideration of the aesthetics or masticatory function (2). Recently, more treatment protocols, including laser surgery and cryosurgery, were used to deal with granulomatous epulis to reserve the teeth and reduce recurrence (3). Each protocol has its advantages. However, incomplete excision (8), continuous secretion of hormone and remaining irritation could also cause the recurrence (3) even in some cases with teeth extraction. In this series, the patients with recurreding granulomatous epulis were all female between 15 and 38-years-old; some patients even had the involved teeth removed. This evidence indicated that hormonal reason may play a critical role in these cases. Furthermore, if surgery is still the treatment of choice to treat, then more extensive resection should be performed, including the removal of more teeth and alveolar bone, which were not accepted by most patients. More important was that more extensive surgery could not confirm success.

A more effective and less aggressive therapy is exigently acquired to treat granulomatous epulis, particularly for recurring lesions. In the present cases, the intralesional PYM injection was used for the relapsed lesions. The exciting result indicated that PYM sclerotherapy may be an additional method for the granulomatous epulis.

PYM is BLM A5 made in China extracted from *Streptomyces Pingyangensisn* with lower pulmonary toxicity than BLM (2,7), which was initially applied in chemotherapy since 1979 (9). The cytotoxicity of PYM is primarily due to DNA damage, as it destroys single- and double-strand DNA, such as chromosomal gaps, deletions and DNA fragmentations (10). Furthermore, PYM has also been widely used as a sclerosing agent for treating infantile haemangiomas, venous (11) and lymphatic (12) malformations because of its high effect and lower incidence of complications . Previous studies indicated that intralesional PYM injection of PYM causes injury and detachment of endothelial cells based on cytotoxicity (7). In Additional, a recent study (13) indicated that an endothelial-mesenchymal transition (EndoMT) in venous malformations was induced by PYM. These effects work together to decrease the number of vascular lumen, thicken the lumen walls and lead to lumen narrowing or occlusion.

Granulomatous epulis share similar histopathological and clinical features with infantile haemangiomas (2). This evidence indicated that the mechanisms of intralesional PYM injection acting in granulomatous epulis may be promoting apoptosis of the endothelial cells and inducing EndoMT of endothelial cells. With regard to the complications, no report on the systemic complications caused by the intralesional PYM injection has yet been published. As a topical medication for injection, complications of PYM are mostly connected with localizsed reactions at the injection site, such as ulceration, pain, local swelling and temporary paraesthesia. In these cases, no systemic complication occurred; the only complaint was local swelling and pain, which were relieved six days after the injection without any intervention (7,12).

Beside PYM, there are also some sclerosing agents which are used for granulomatous epulis or oral pyogenic granuloma, including absolute ethanol (14), sodium tetradecyl sulfate (STS) sclerotherapy (15) and corticosteroid (16). Although these sclerosing agents have been proven to be effective, the application limitations should not be ignored. Ethanol is restrictedly used because some patients have an allergy to alcohol and its injection may cause severe soft tissue oedema (17). Infiltrations of STS into stromal tissues induce nonspecial necrotic changes (3). Moreover, local nerve injury was reported after STS sclerotherapy of venous malformations (18).

Taken together, intralesional PYM injection was safe and highly effective in the treatment of granulomatous epulis. In the present cases, the lesions were completely involuted by PYM with no recurrence and no tissue destruction, whilereas the teeth adjacent to the lesions were reserved. This exciting result indicates that PYM sclerotherapy may become a preferred treatment modality for relapsing granulomatous epulis.
